# Nonsynonymous single-nucleotide polymorphisms in the *G6PC2* gene affect protein expression, enzyme activity, and fasting blood glucose

**DOI:** 10.1016/j.jbc.2021.101534

**Published:** 2021-12-23

**Authors:** Emily M. Overway, Karin J. Bosma, Derek P. Claxton, James K. Oeser, Kritika Singh, Lindsay B. Breidenbach, Hassane S. Mchaourab, Lea K. Davis, Richard M. O'Brien

**Affiliations:** 1 Department of Molecular Physiology and Biophysics, Vanderbilt University School of Medicine, Nashville, Tennessee, USA; 2 Department of Medicine, Vanderbilt University School of Medicine, Nashville, Tennessee, USA

**Keywords:** islet, glucose metabolism, insulin secretion, single-nucleotide polymorphism (SNP), enzyme degradation, glucose-6-phosphatase, AA, amino acid, EHR, electronic health record, ER, endoplasmic reticulum, FBG, fasting blood glucose, G6Pase, glucose-6-phosphatase, G6PC, glucose-6-phosphatase catalytic subunit, GSD1a, glycogen storage disease type 1a, GSIS, glucose-stimulated insulin secretion, GWAS, genome-wide association studies, HRP, horseradish peroxidase, MAF, minor allele frequency, ORF, open reading frame, SNP, single-nucleotide polymorphism, WT, wild-type

## Abstract

*G6PC2* encodes a glucose-6-phosphatase (G6Pase) catalytic subunit that modulates the sensitivity of insulin secretion to glucose and thereby regulates fasting blood glucose (FBG). A common single-nucleotide polymorphism (SNP) in *G6PC2*, rs560887 is an important determinant of human FBG variability. This SNP has a subtle effect on *G6PC2* RNA splicing, which raises the question as to whether nonsynonymous SNPs with a major impact on G6PC2 stability or enzyme activity might have a broader disease/metabolic impact. Previous attempts to characterize such SNPs were limited by the very low inherent G6Pase activity and expression of G6PC2 protein in islet-derived cell lines. In this study, we describe the use of a plasmid vector that confers high G6PC2 protein expression in islet cells, allowing for a functional analysis of 22 nonsynonymous G6PC2 SNPs, 19 of which alter amino acids that are conserved in mouse G6PC2 and the human and mouse variants of the related G6PC1 isoform. We show that 16 of these SNPs markedly impair G6PC2 protein expression (>50% decrease). These SNPs have variable effects on the stability of human and mouse G6PC1, despite the high sequence homology between these isoforms. Four of the remaining six SNPs impaired G6PC2 enzyme activity. Electronic health record–derived phenotype analyses showed an association between high-impact SNPs and FBG, but not other diseases/metabolites. While homozygous *G6pc2* deletion in mice increases the risk of hypoglycemia, these human data reveal no evidence that the beneficial use of partial G6PC2 inhibitors to lower FBG would be associated with unintended negative consequences.

Glucose-6-phosphatase catalyzes the hydrolysis of glucose-6-phosphate (G6P) to glucose and inorganic phosphate and is located in the endoplasmic reticulum (ER) membrane (1, 2). It exists as a multicomponent enzyme system in which a G6P transporter, encoded by the *SLC37A4* gene, delivers substrate from the cytosol to the active site of a glucose-6-phosphatase catalytic subunit (G6PC) in the lumen of the ER, with transporters for inorganic phosphate and glucose returning the reaction products back to the cytosol (1, 2). The *G6PC* gene family comprises three members, namely *G6PC1*, *G6PC2,* and *G6PC3* (1). *G6PC1*, also known as *G6Pase* and *G6PC*, is predominantly expressed in the liver, kidney, and intestine where it catalyzes the terminal step in the gluconeogenic and glycogenolytic pathways, whereas *G6PC3*, also known as *UGRP*, is widely expressed, with especially high expression in the kidney, testis, skeletal muscle, and brain (1). *G6PC2*, also known as *IGRP*, is predominantly expressed in pancreatic islets where, in humans, expression is approximately fivefold higher in beta than alpha cells (3, 4, 5).

In conjunction with the beta cell glucose sensor, glucokinase, G6PC2 acts to create a futile substrate cycle that fine-tunes the sensitivity of glucose-stimulated insulin secretion (GSIS) to glucose by modulating the rate of beta cell glycolytic flux (6, 7, 8, 9). This futile cycle contributes to the ability of beta cells to regulate fasting blood glucose (FBG) (6, 7, 8, 9). Key evidence in support of this model are the observations that, in isolated *G6pc2* knockout (KO) islets, glucose-6-phosphatase activity (8) and glucose cycling (10) are both reduced, whereas glycolysis is elevated (9). Deletion of *G6pc2* results in a leftward shift in the dose–response curve for GSIS (8) such that under fasting conditions, insulin levels are the same in wild-type (WT) and *G6pc2* KO mice, but FBG is reduced in KO mice (8, 9, 11, 12). These observations are consistent with genome-wide association studies (GWAS) and molecular studies that have linked the rs560887 “A” allele to reduced *G6PC2* expression and reduced FBG (13, 14, 15, 16).

Experiments in mice suggest that the nature of the physiological benefits associated with G6PC2 are that it confers a transient, beneficial elevation in FBG during periods of stress (17, 18) and that it protects against hypoglycemia in response to a ketogenic diet or prolonged fasting (19). Previous biobank analyses have confirmed the association between *G6PC2* and FBG and have also linked *G6PC2* to an altered risk for acute pancreatitis (9). However, a caveat with these studies is that they were performed using the rs560887 *G6PC2* SNP that has a mild effect on *G6PC2* RNA splicing (16) such that other deleterious consequences of altered *G6PC2* expression might have been missed. The present studies addressed that caveat by first identifying nonsynonymous *G6PC2* SNPs that have a major effect on G6PC2 protein expression or activity in islet cells and then assessing the impact of these SNPs on human health through biobank analyses.

## Results

### G6PC2 protein expression is enhanced using the pJPA5 plasmid

**Figure 1:**
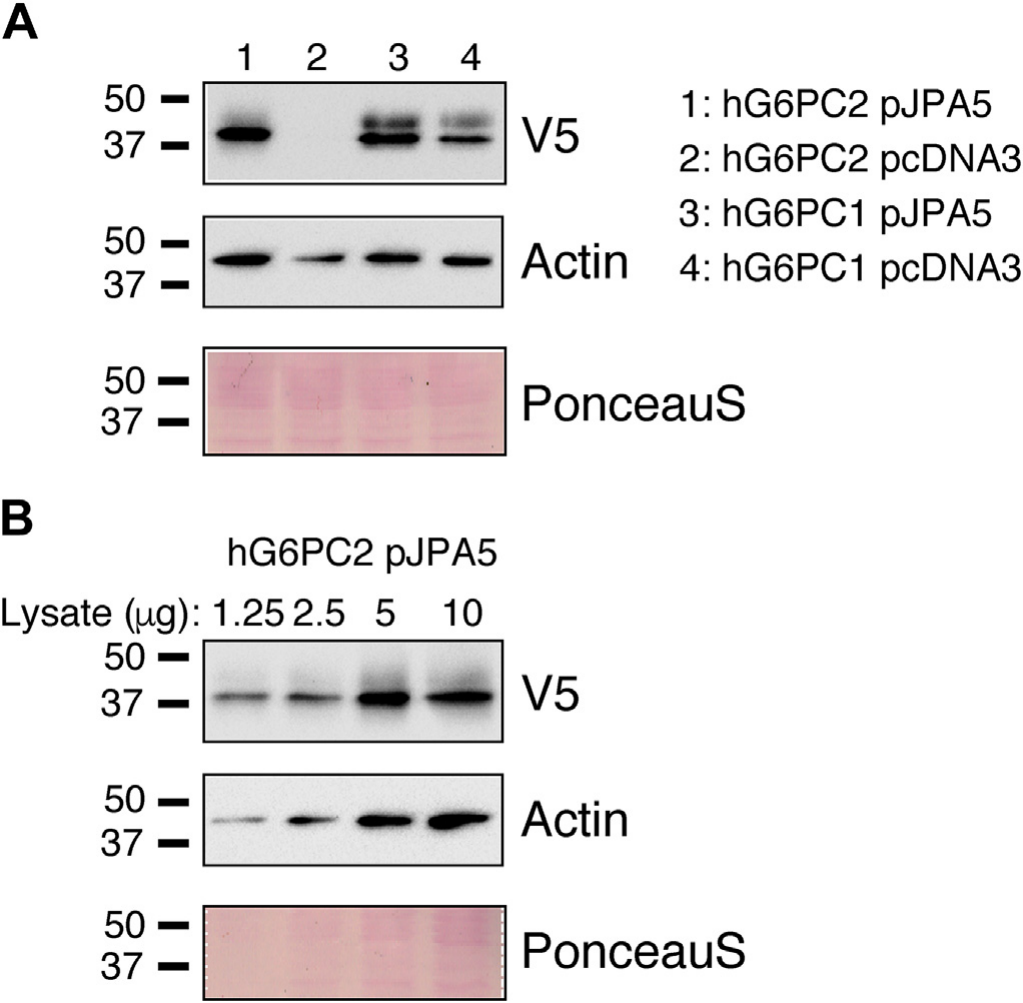
**Comparison of human G6PC1 and G6PC2 protein expression driven by the pcDNA3.1D and pJPA5 vectors.** 832/13 cells were transiently transfected with either pcDNA3.1D or pJPA5 expression vectors encoding human G6PC1 or G6PC2 with a C terminal V5 His Tag. Following transfection, cells were incubated for 18–20 h in serum-containing media. Cells were subsequently harvested and protein expression assayed by Western blotting as described in Experimental procedures. *A,* G6PC expression was assessed using an anti-V5 antibody and equal protein loading was confirmed using both Ponceaus staining and measurement of actin expression. *B,* the conditions used to assess G6PC2 and actin expression are semiquantitative. Representative blots are shown. G6PC, glucose-6-phosphatase catalytic subunit.

Analyses of the functional effects of nonsynonymous *G6PC2* SNPs have been limited by an inability to drive high G6PC2 protein expression in islet-derived cell lines, preventing the detection of G6PC2 enzyme activity *in vitro* (3, 4, 20, 21, 22). To overcome this limitation, we compared the level of human G6PC2 protein expression driven by a number of plasmid vectors in the islet-derived 832/13 cell line (23). Western blotting analyses demonstrated that enhanced expression of G6PC2 protein could be achieved when the human *G6PC2* open reading frame (ORF) with a V5 C terminal tag was ligated into the pJPA5 vector (24) (Fig. 1*A*). Under the experimental conditions used, these Western blotting assays were semiquantitative (Fig. 1*B*) (Fig. S1). The pJPA5 vector contains additional cytomegalovirus (CMV) 5′ untranslated region and linker sequence relative to the pcDNA3.1D vector used in our initial experiments (Fig. S2). In contrast to G6PC2, when the human *G6PC1* ORF was ligated into the pJPA5 plasmid, G6PC1 protein expression was only enhanced modestly relative to that achieved with the pcDNA3.1D vector (Fig. 1*A*). Similar levels of G6PC1 and G6PC2 protein expression were achieved using the pJPA5 vector (Fig. 1*A*).

### Multiple nonsynonymous *G6PC2* SNPs alter G6PC2 protein expression

**Figure 2:**
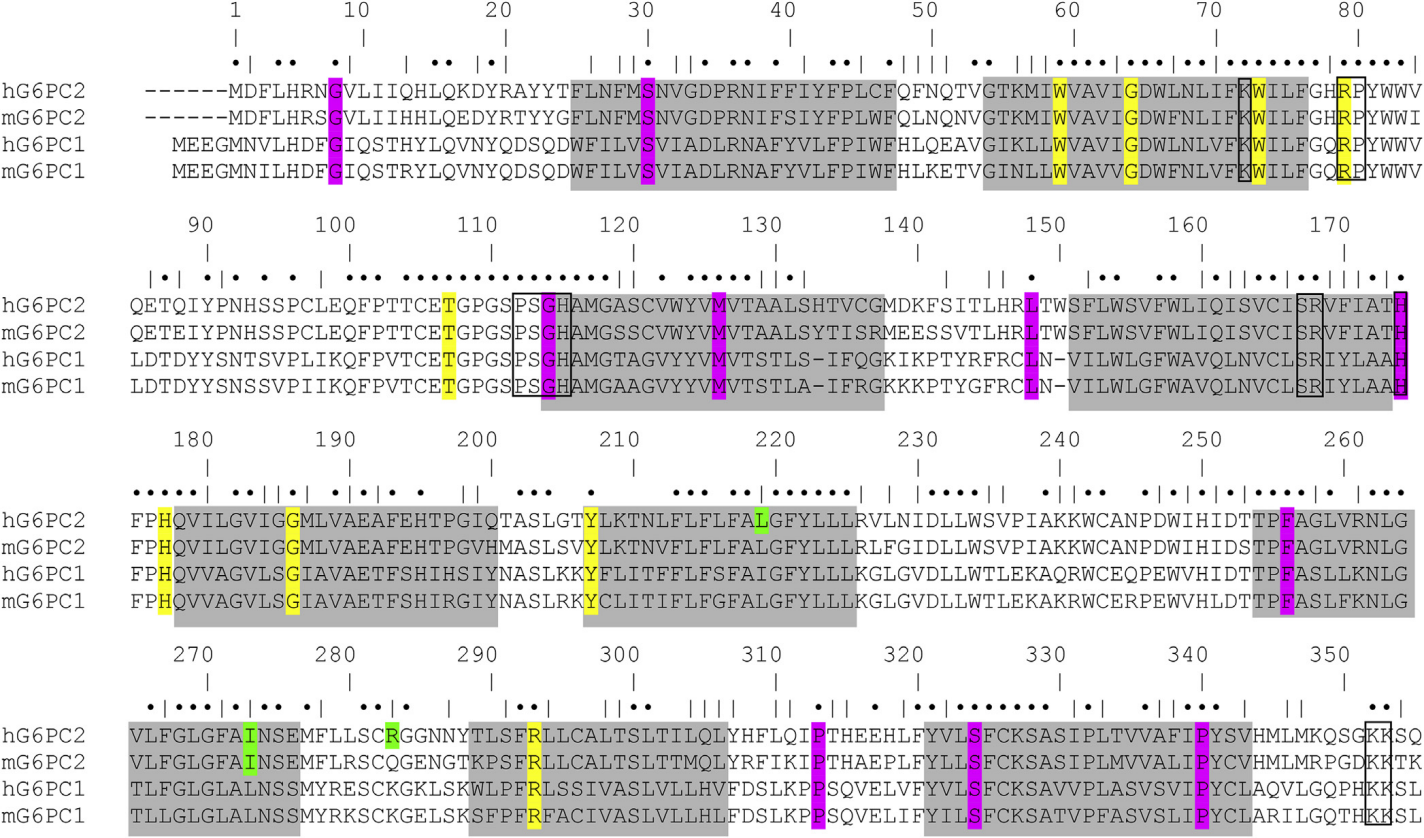
**Conservation of amino acids between human G6PC2, mouse G6PC2, human G6PC1, and mouse G6PC1.** Sequence alignment showing the conservation of AAs between human (h) and mouse (m) G6PC1 and G6PC2. Residues highlighted in *yellow* represent conserved AAs that are changed by a human *G6PC2* SNP and where mutation in G6PC1 can cause GSD type 1a (26) (Table 1). Residues highlighted in *pink* represent conserved AAs that are changed by human *G6PC2* SNPs but where mutation in G6PC1 has not been associated with GSD type 1a. Residues highlighted in *green* represent nonconserved AAs that are changed by human *G6PC2* SNPs. Identities are indicated by *filled circles* and similarities by vertical bars. *Gray boxes* represent putative transmembrane domains determined using the TMHMM algorithm (https://services.healthtech.dtu.dk/) (60). The predictions made by this program differ from earlier models (4, 61). The figure shows the predicted domains for human G6PC1. G6PC1 and G6PC2 have an extended sequence motif (KXXXXXXRP-(X12-54)-PSGH-(X31-54)-SRXXXXXHXXXD) (*boxed*), similar to that found in bacterial vanadate-sensitive haloperoxidases and mammalian phosphatidic acid phosphatases, which constitutes the active site of these enzymes (62). G6PC1 and G6PC2 also share a C terminal ER retention sequence (KK; *boxed*) (63). Adapted from Ref. (22). G6PC, glucose-6-phosphatase catalytic subunit; GSD1a, glycogen storage disease type 1a; SNP, single-nucleotide polymorphism.

We previously attempted to circumvent the prior limitations associated with low G6PC2 protein overexpression in islet-derived cell lines by indirectly examining the effects of 22 nonsynonymous *G6PC2* SNPs that alter amino acids that are conserved between mouse and human G6PC1 and G6PC2, on mouse G6PC1 protein expression and enzyme activity in 832/13 cells, and/or human G6PC2 protein expression in COS cells (22). This approach exploited the much greater catalytic activity of G6PC1 *versus* G6PC2 (25), but a major caveat is the fact that G6PC1 and G6PC2 share only 50% sequence identity (Fig. 2), so even mutations of conserved amino acids (AAs) may have differential effects on G6PC1 and G6PC2 activity and stability. In addition, COS cells, a fibroblast-like cell line derived from kidney tissue, may lack factors in islets that impact protein folding or stability, alter interactions between G6PC2 and other proteins, or alter posttranslational modifications, all of which could affect the impact of specific SNPs.

Of the 22 SNPs previously examined using this indirect approach, nine of them (Gln16His, Val53Ile, Asn68Ile, Tyr124Cys, Ser203Arg, Phe215Ser, Tyr222His, His250Tyr, and Leu301Ser) resulted in a less than 15% change in mouse G6PC1 activity in a novel, intact cell glucose-6-phosphatase assay (22), and so were not reanalyzed in this study. The remaining 13 *G6PC2* SNPs either impaired mouse G6PC1 activity more than 15% or were associated with reduced human G6PC2 protein expression in COS cells (Gln8Glu, Ser30Phe, Arg79Gln, Thr107Arg, Gly114Arg, Met126Val, His177Tyr, Tyr207Ser, Phe256Leu, Arg293Trp, Pro313Leu, Ser324Pro, and Pro340Leu) (22).

Having now achieved relatively high expression of human G6PC2 protein in an islet-derived cell line using the pJPA5 vector, we reanalyzed the direct effects of these 13 SNPs on G6PC2 protein expression and activity. We also analyzed an additional six nonsynonymous *G6PC2* SNPs (Trp59Arg, Gly64Arg, Trp73Arg, Leu148Pro, His174Tyr, and Gly186Asp) that were identified in various databases (see Experimental procedures) since our previous study. These SNPs also affect AAs that are conserved in mouse and human G6PC1 and mouse G6PC2 (Fig. 2). We hypothesized that the conservation of these 19 AAs indicated that they were likely to be functionally important. In addition, we also analyzed three additional SNPs, which affect AAs that are not conserved in mouse or human G6PC1. Two are missense changes (Leu219Val and Ile273Val) (Fig. 2) and one generates a termination mutation (Arg283STOP) lacking the C terminal 72 AAs. We hypothesized that studying the functional effect of these three variants was important because they have relatively high minor allele frequencies (MAFs). Therefore, if they were found to affect G6PC2 function, they would likely be amenable to further analysis using human biobanks linked to medical records.

**Figure 3:**
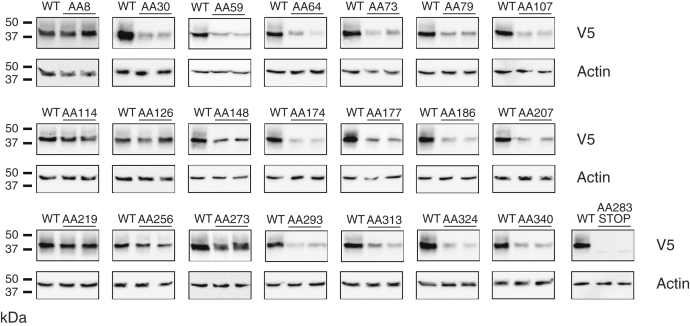
**Analysis of the effect of human *G6PC2* SNPs on G6PC2 protein expression.** 832/13 cells were transiently transfected with pJPA5 expression vectors encoding human G6PC2 with a C terminal V5 His Tag. Following transfection, cells were incubated for 18–20 h in serum-containing media. Cells were subsequently harvested and protein expression assayed by Western blotting as described in Experimental procedures. G6PC2 expression was assessed using an anti-V5 antibody and equal protein loading was confirmed by measurement of actin expression. Representative blots are shown. G6PC, glucose-6-phosphatase catalytic subunit; SNP, single-nucleotide polymorphism.

**Figure 4:**
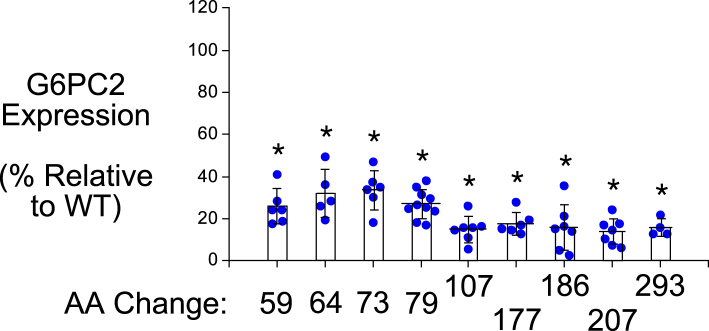
**Analysis of the effect of human *G6PC2* SNPs that affect conserved amino acids associated with GSD1a in the context of human G6PC1, on G6PC2 protein expression.** Human *G6PC2* SNPs were identified that affect AAs conserved between mouse and human G6PC1 and mouse and human G6PC2 where a mutation is known to cause GSD1a in the context of human G6PC1 (Table 1). The effect of these *G6PC2* SNPs on G6PC2 protein expression was determined as described in Fig. 3. Protein expression was quantified as described in Experimental procedures. Results show mean data −/+ SD, n = 4–10. ∗*p* < 0.05 *vs.* WT. G6PC, glucose-6-phosphatase catalytic subunit; SNP, single-nucleotide polymorphism.

For a subset of 9 of the 19 SNPs that affect AAs conserved in mouse and human G6PC1, mutation of these AAs in human G6PC1 has been shown to cause glycogen storage disease type 1a (GSD1a) (26), directly demonstrating that they are functionally important (Fig. 2; Table 1). We assessed the impact of these nine human *G6PC2* SNPs on G6PC2 protein expression in 832/13 cells using Western blotting (Figs. 3 and 4) (Table 1). All nine AA changes were associated with a substantial (>50%) reduction in G6PC2 protein expression (Figs. 3 and 4) (Table 1).

**Table 1:** Analysis of the effect of human *G6PC2* SNPs that affect conserved amino acids associated with GSD1a in the context of human G6PC1

*G6PC2* SNP	G6PC2 AA#	Mutation in human G6PC1 causing GSD1a	Domain Location	G6PC2 protein expression (%WT)	G6PC2 SIFT prediction	G6PC1 AA#	G6PC1 protein expression (%WT)
rs369755574	Trp59Arg	Arg	TM 2	25.9 ± 8.5^a^	Deleterious	63	1.7 ± 1.1^a^
rs762205787	Gly64Arg	Arg	TM 2	32.0 ± 11.4^a^	Deleterious	68	1.9 ± 1.9^a^
rs756028690	Trp73Arg	Arg	TM 2	33.7 ± 9.3^a^	Deleterious	77	25.7 ± 7.1^a^
rs144254880	Arg79Gln	His or Cys	Loop	27.1 ± 6.8^a^	Deleterious	83	50.7 ± 23.8^a^
rs371234742	Thr107Arg	Ile	Loop	15.2 ± 6.2^a^	Deleterious	111	3.6 ± 3.8^a^
rs138726309	His177Tyr	Pro	Loop	17.9 ± 5.4^a^	Deleterious	179	30.2 ± 19.0^a^
rs764817338	Gly186Asp	Asp or Arg or Ser	TM 5	15.9 ± 10.9^a^	Deleterious	188	26.5 ± 14.0^a^
rs2232323	Tyr207Ser	Cys	TM 6	14.1 ± 6.3^a^	Deleterious	209	12.7 ± 8.8^a^
rs374055555	Arg293Trp	Cys	TM 8	16.0 ± 4.3^a^	Deleterious	295	5.5 ± 0.5^a^

Human *G6PC2* SNPs were identified that affect amino acids (AAs) conserved between mouse and human G6PC1 and mouse and human G6PC2 where a mutation is known to cause GSD1a in the context of human G6PC1. The amino acid change associated with GSD1a is shown as are the domain location and the effect of the AA change as predicted using the SIFT algorithm (64). The location of putative transmembrane domains for human G6PC1 were determined using the TMHMM algorithm (https://services.healthtech.dtu.dk/) (60). The predictions made by this program differ from earlier models (4, 61). Protein expression was quantified as described in Experimental procedures. Results show mean data ± SD, n = 4–10.

TM, transmembrane domain.

a *p* < 0.05 vs. WT.

**Figure 5:**
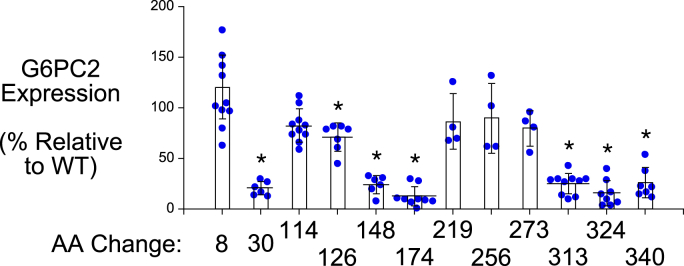
**Analysis of the effect of human G6PC2 SNPs that affect amino acids that are not associated with GSD1a in the context of human G6PC1, on G6PC2 protein expression.** Human *G6PC2* SNPs were identified that affect AAs conserved between mouse and human G6PC1 and mouse and human G6PC2, which are not associated with GSD1a in the context of human G6PC1, as well as amino acids that are not conserved in G6PC1 (Table 2). The effect of these *G6PC2* SNPs on G6PC2 protein expression was determined as described in Fig. 3. Protein expression was quantified as described in Experimental procedures. Results show mean data −/+ SD, n = 4 to 10. ∗*p* < 0.05 *vs.* WT. G6PC, glucose-6-phosphatase catalytic subunit; SNP, single-nucleotide polymorphism.

Next, we examined the impact of the ten human *G6PC2* SNPs that affect AAs that are conserved in human and mouse G6PC1 but are not associated with GSD1a, on human G6PC2 protein expression in 832/13 cells. Six of these ten variants (Ser30Phe, Leu148Pro, His174Tyr, Pro313Leu, Ser324Pro, and Pro340Leu) resulted in a substantial (>50%) decrease in G6PC2 protein expression (Figs. 3 and 5) (Table 2). The other four variants (Gly8Glu, Gly114Arg, Met126Val, Phe256Leu) resulted in little (<35%) to no change in protein expression (Figs. 3 and 5) (Table 2).

**Table 2:** Analysis of the effect of human *G6PC2* SNPs that affect amino acids that are not associated with GSD1a in the context of human G6PC1

*G6PC2* SNP	G6PC2 AA#	Domain location	G6PC2 protein expression (%WT)	G6PC2 SIFT Prediction	G6PC1 AA#	G6PC1 protein expression (%WT)
rs368382511	Gly8Glu	NH2 terminus	121.0 ± 31.6	Deleterious	12	43.0 ± 18.7^a^
rs142189264	Ser30Phe	TM 1	21.1 ± 6.8^a^	Deleterious	34	18.0 ± 10.6^a^
rs149663725	Gly114Arg	TM3	82.8 ± 16.3	Deleterious	118	90.9 ± 30.8
rs367930047	Met126Val	TM 3	71.3 ± 13.7^a^	Deleterious	130	54.8 ± 20.6^a^
rs761866606	Leu148Pro	Loop	24.5 ± 9.4^a^	Deleterious	151	46.7 ± 24.5^a^
rs746984825	His174Tyr	Loop	13.2 ± 9.7^a^	Deleterious	176	31.0 ± 16.7^a^
rs492594	Leu219Val	TM 6	86.9 ± 27.2	Tolerated	221 (Ile)	N.A.
rs150538801	Phe256Leu	TM7	90.0 ± 34.1	Deleterious	258	67.6 ± 17.7^a^
rs148689354	Ile273Val	TM7	80.1 ± 17.2	Deleterious	275 (Leu)	N.A.
rs137857125	Pro313Leu	Loop	25.6 ± 10.2^a^	Deleterious	315	11.2 ± 8.0^a^
rs2232326	Ser324Pro	TM 9	16.1 ± 12.8^a^	Deleterious	326	18.5 ± 11.4^a^
rs2232327	Pro340Leu	TM 9	26.5 ± 14.9^a^	Deleterious	342	5.2 ± 2.6^a^
rs146779637	Arg283STOP	Loop	N.D.	N.A.	285 (Lys)	N.A.

Human *G6PC2* SNPs were identified that affect amino acids conserved between mouse and human G6PC1 and mouse and human G6PC2 that are not associated with GSD1a in the context of human G6PC1, as well as amino acids that are not conserved in G6PC1. The domain location and the effect of the AA change as predicted using the SIFT algorithm (64) are shown. The location of putative transmembrane domains for human G6PC1 were determined using the TMHMM algorithm (https://services.healthtech.dtu.dk/) (60). The predictions made by this program differ from earlier models (4, 61). Protein expression was quantified as described in Experimental procedures. Results show mean data ±SD, n = 4–10.

N.A., not applicable; N.D., not detected; TM, transmembrane domain.

a *p* < 0.05 vs. WT.

Finally, we analyzed the impact of the termination mutation (Arg283STOP) and the two additional SNPs (Leu219Val and Ile273Val) that affect AAs that are not conserved in mouse or human G6PC1 (Fig. 2) on human G6PC2 protein expression in 832/13 cells. Expression of the truncated variant was markedly reduced (Fig. 3). Neither the Val219Leu nor Ile273Val SNPs resulted in a marked change in G6PC2 protein expression (Figs. 3 and 5) (Table 2).

### Identification of nonsynonymous G6PC2 SNPs that alter G6PC2 activity *in vitro*

**Figure 6:**
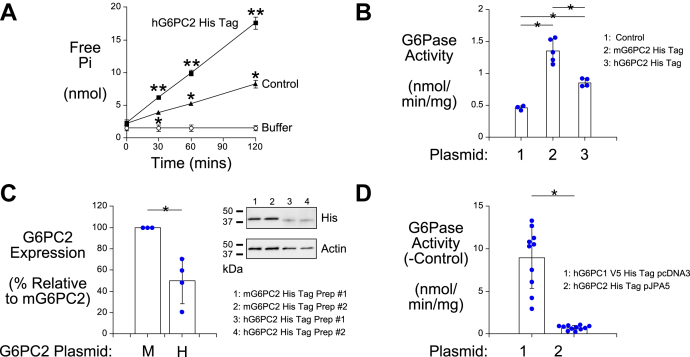
**Analysis of G6PC2 glucose-6-phosphatase (G6Pase) activity.***A, B,* and *D,* 832/13 cells were transfected with pJPA5 or pcDNA3.1D expression vectors encoding the proteins shown, microsomes were isolated and G6Pase activity was measured as described in Experimental procedures. In (*A*) results show mean data −/+ SD, n = 3–4. ∗*p* < 0.05 Control *vs.* Buffer, ∗∗*p* < 0.05 G6PC2 *vs.* Control, two-way ANOVA. Using data collected at the t = 120 time point, mean G6PC2 and Control G6Pase activity −/+ SD were 8.36 ± 0.67 and 4.04 ± 0.13 nmol/min/mg, respectively. In (*B*) results show mean data −/+ SD, n = 3–5. Statistical comparisons were made using one-way ANOVA with a Tukey post-hoc test; ∗*p* < 0.05. In (*D*) results show mean data −/+ SD, n = 10–11. Statistical comparisons were made using a *t* test; ∗*p* < 0.05. *C,* 832/13 cells were transiently transfected with pJPA5 expression vectors encoding G6PC2 as shown. Following transfection, cells were incubated for 18–20 h in serum-containing media. Cells were subsequently harvested and protein expression assayed by Western blotting as described in Experimental procedures. G6PC2 expression was assessed using an anti-His antibody and equal protein loading was confirmed by measurement of actin expression. Representative blots are shown. Statistical comparisons were made using a *t* test; ∗*p* < 0.05. G6Pase, glucose-6-phosphatase; G6PC, glucose-6-phosphatase catalytic subunit.

For the six variants that have little to no effect on G6PC2 protein expression (Gly8Glu, Gly114Arg, Met126Val, Val219Leu, Phe256Leu, and Ile273Val) (Figs. 3 and 5) (Table 2), we considered the possibility that they might instead affect G6PC2 enzyme activity. To address this possibility, we directly measured glucose-6-phosphatase (G6Pase) activity *in vitro* using microsomal membranes prepared from 832/13 cells that had been transiently transfected with plasmids encoding these G6PC2 variants. Our previous attempts at detecting microsomal G6Pase activity *in vitro* following transient expression of G6PC2 in various cell lines had been unsuccessful; however, these studies all used the pcDNA3.1D vector (3, 4, 20, 21). With the much higher G6PC2 protein expression achieved with the pJPA5 vector (Fig. 1*A*), low microsomal G6Pase activity could now be detected *in vitro* following transient expression of G6PC2 in 832/13 cells (Fig. 6*A*). *G6pc2* is a pseudogene in rats such that endogenous G6PC2 activity is absent in rat 832/13 cells (4). The background activity in the G6Pase assay (Fig. 6*A*) is therefore due to an unknown phosphatase. Higher G6Pase activity was detected following transient expression of His tagged mouse G6PC2 compared with His-tagged human G6PC2 (Fig. 6*B*), though this was largely due to the higher protein expression of mouse G6PC2 (Fig. 6*C*). Figure 6*D* shows that when the pcDNA3.1D vector was used to drive G6PC1 protein expression to a similar level as G6PC2 protein expression achieved with the pJPA5 vector (Fig. 1*A*), G6PC1 exhibited much higher G6Pase activity than G6PC2 in these *in vitro* assays (Fig. 6*D*).

**Figure 7:**
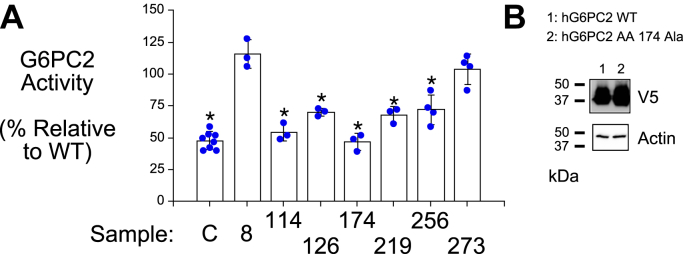
**Analysis of the effect of human *G6PC2* SNPs on G6PC2 G6Pase activity.***A,* 832/13 cells were transfected with pJPA5 expression vectors encoding the WT or variant G6PC2 His Tag proteins shown, microsomes were isolated and G6Pase activity was measured as described in Experimental procedures. Results show mean data −/+ SD, n = 3–8. Statistical comparisons with WT were made using T tests; ∗*p* < 0.05. C, control lysate. *B,* 832/13 cells were transiently transfected with pJPA5 expression vectors encoding WT or catalytically dead G6PC2 (AA 174 Ala) as shown. Following transfection, cells were incubated for 18–20 h in serum-containing media. Cells were subsequently harvested and protein expression assayed by Western blotting as described in Experimental procedures. G6PC2 expression was assessed using an anti-V5 antibody and equal protein loading was confirmed by measurement of actin expression. Representative blots are shown. G6Pase, glucose-6-phosphatase; G6PC, glucose-6-phosphatase catalytic subunit.

We previously showed that conversion of AA 176, the phosphate acceptor site in G6PC1 (27), from a histidine to an alanine, does not affect G6PC1 protein expression (22), though it abolishes phosphatase activity (27). Similarly, mutation of the equivalent AA in G6PC2, histidine 174, abolished phosphatase activity (Fig. 7*A*) without affecting G6PC2 protein expression (Fig. 7*B*). The *in vitro* G6Pase assay showed that the Gly8Glu and Ile273Val variants did not affect activity, whereas the Gly114Arg, Met126Val, Val219Leu, and Phe256Leu variants were associated with reduced activity (Fig. 7*A*). For the Gly114Arg, Met126Val, and Phe256Leu variants, these results are consistent with the SIFT analyses that predicted these variants would be deleterious (Table 2). In contrast, the effects, or lack thereof, of the Gly8Glu, Ile273Val, and Leu219Val variants (Fig. 7*A*) were at odds with the SIFT predictions (Table 2).

### Analysis of nonsynonymous G6PC2 SNPs that alter G6PC2 activity *in situ*

**Figure 8:**
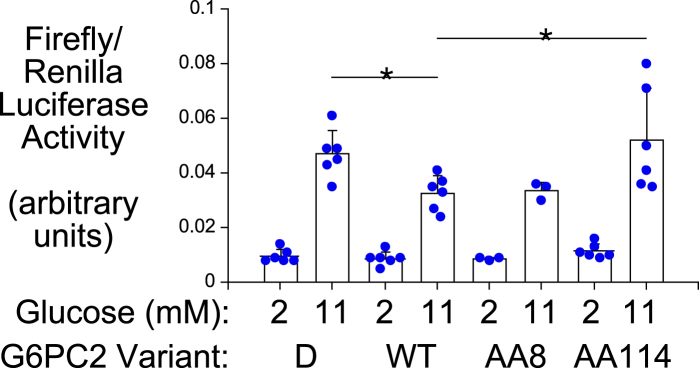
**Analysis of the suppression of glucose-stimulated fusion gene expression by G6PC2 in 832/13 cells.** 832/13 cells were transiently cotransfected with the −7248/+62 *G6pc1*-*luciferase* fusion gene (2 μg), an expression vector encoding *Renilla* luciferase (0.5 μg) and expression vectors (1 μg) encoding either wild type (WT), catalytically dead (D; AA 174 Ala), or variant (AA8, AA114) G6PC2. Following transfection, cells were incubated for 18 to 20 h in serum-free medium in the presence of 2 or 11 mM glucose. Cells were then harvested and luciferase activity assayed as described in Experimental procedures. Results show mean data −/+ SD (n = 3–6) and were calculated as the ratio of firefly:*Renilla* luciferase activity. Statistical comparisons to 11 mM WT were made using ANOVA with a Dunnett's Multiple Comparisons post-hoc test; ∗*p* < 0.05. G6PC, glucose-6-phosphatase catalytic subunit.

Chou and colleagues (28) have shown that G6PC1 appears to couple to the SLC37A4 G6P transporter, resulting in the activation of G6P transport. The mechanism is unknown, and no evidence exists for a physical interaction between G6PC1 and SLC37A4. However, we considered the possibility that the Gly8Glu variant, which did not affect G6PC2 activity *in vitro* (Fig. 7*A*), might affect an interaction between G6PC2 and SLC37A4 that activated G6P transport and hence G6PC2 activity. To address this possibility, we used an *in situ* assay previously developed by our lab to study the effect of G6PC1 mutations on G6PC1 enzyme activity in intact cells (22). This transient transfection assay uses a combination of a glucose-responsive *G6pc1*-*luciferase* fusion gene and the rat 832/13 cell line. We previously showed that luciferase activity is induced by elevated glucose in this assay but repressed by cotransfection with a plasmid encoding G6PC1 (22). We hypothesized that G6PC1 reduces intracellular G6P, the key metabolite in glucose-regulated gene transcription (22, 29). Again, taking advantage of the high G6PC2 protein expression conferred by the pJPA5 vector, this assay was used to study G6PC2 activity in intact cells. Luciferase activity was induced by elevated glucose but repressed by cotransfection with a plasmid encoding G6PC2 (Fig. 8). Cotransfection with the catalytically dead G6PC2 AA 174 variant was used to control for ER stress (Fig. 8). Using this assay, we observed that the G6PC2 Gly114Arg variant abolished G6Pase activity *in situ* (Fig. 8) as it did *in vitro* (Fig. 7*A*), whereas the G6PC2 Gly8Glu variant still repressed luciferase activity (Fig. 8), indicating that it does not disrupt a putative activating interaction between G6PC2 and SLC37A4.

### A subset of nonsynonymous *G6PC2* SNPs alter G6PC1 protein expression

**Figure 9:**
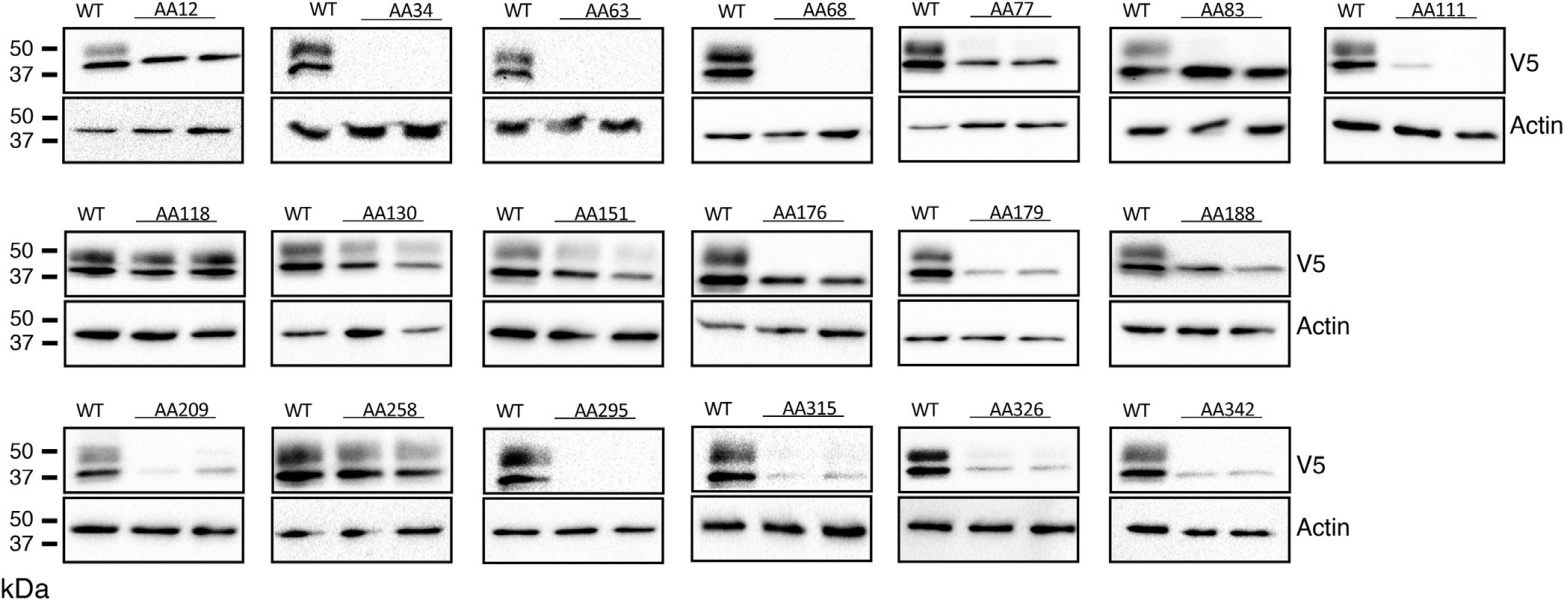
**Analysis of the effect of human *G6PC2* SNPs on G6PC1 protein expression.** 832/13 cells were transiently transfected with pcDNA3.1D expression vectors encoding human G6PC1 with a C terminal V5 His Tag. Following transfection, cells were incubated for 18–20 h in serum-containing media. Cells were subsequently harvested and protein expression assayed by Western blotting as described in Experimental procedures. G6PC1 expression was assessed using an anti-V5 antibody and equal protein loading was confirmed by measurement of actin expression. Representative blots are shown. G6PC, glucose-6-phosphatase catalytic subunit; SNP, single-nucleotide polymorphism.

A surprising aspect of the analysis of *G6PC2* SNPs that affect AAs conserved between G6PC1 and G6PC2 was the number that showed reduced G6PC2 protein expression (Tables 1 and 2). Similar analyses of mutations in *G6PC1* that result in GSD1a suggest that the majority mainly affect enzyme activity rather than G6PC1 protein expression (26, 30). To explore the hypothesis that G6PC2 protein expression is more sensitive to the effects of mutations than G6PC1, we repeated the analysis of the 19 SNPs that affect AAs conserved between G6PC1 and G6PC2 by analyzing their effect on human G6PC1 protein expression in 832/13 cells using Western blotting (Fig. 9).

**Figure 10:**
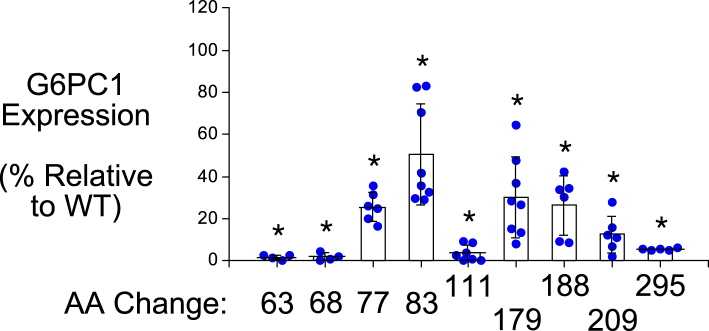
**Analysis of the effect of human *G6PC2* SNPs that affect conserved amino acids associated with GSD1a in the context of human G6PC1, on G6PC1 protein expression.** Human *G6PC2* SNPs were identified that affect AAs conserved between mouse and human G6PC1 and mouse and human G6PC2 where a mutation is known to cause GSD1a in the context of human G6PC1 (Table 1). The effect of these *G6PC2* SNPs on G6PC1 protein expression was determined as described in Fig. 3. Protein expression was quantified as described in Experimental procedures. Results show mean data −/+ SD, n = 4–8. ∗*p* < 0.05 *vs.* WT. G6PC, glucose-6-phosphatase catalytic subunit; SNP, single-nucleotide polymorphism.

**Figure 11:**
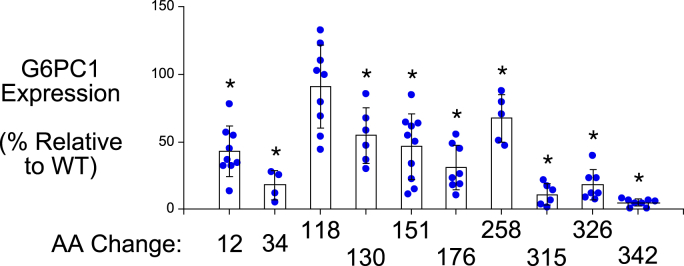
**Analysis of the effect of human G6PC2 SNPs that affect amino acids that are not associated with GSD1a in the context of human G6PC1, on G6PC1 protein expression.** Human *G6PC2* SNPs were identified that affect AAs conserved between mouse and human G6PC1 and mouse and human G6PC2, that are not associated with GSD1a in the context of human G6PC1 (Table 2). The effect of these *G6PC2* SNPs on G6PC1 protein expression was determined as described in Fig. 3. Protein expression was quantified as described in Experimental procedures. Results show mean data −/+ SD, n = 4–10. ∗*p* < 0.05 *vs.* WT. G6PC, glucose-6-phosphatase catalytic subunit; SNP, single-nucleotide polymorphism.

Table 1 and Figure 10 show the impact of the nine *G6PC2* SNPs that affect AAs whose mutation in human G6PC1 has been shown to cause GSD1a. Table 2 and Figure 11 show the impact of the ten *G6PC2* SNPs that affect AAs that are conserved in human G6PC1 but are not associated with GSD1a. Overall these results do not support the hypothesis that G6PC2 stability is more sensitive to the effects of mutations than G6PC1. Instead, a more complex pattern emerged in which certain mutations had a greater effect on G6PC1 stability than G6PC2 and vice versa (Tables 1 and 2) (Fig. S3).

**Figure 12:**
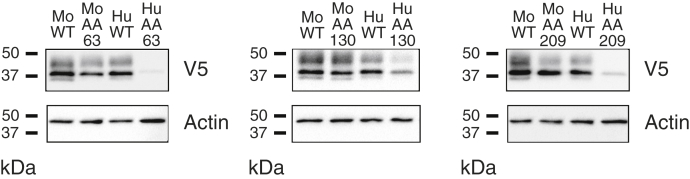
**Comparison of the effect of specific amino acid changes on the expression of mouse and human G6PC1.** Human *G6PC2* SNPs were identified that affect AAs conserved between mouse and human G6PC1 and mouse and human G6PC2 (Tables 1 and 2). The effect of these *G6PC2* SNPs on mouse and human G6PC1 protein expression was determined as described in Fig. 3. A representative blot is shown. G6PC, glucose-6-phosphatase catalytic subunit.

Interestingly, a comparison of these results with our published data examining the effect of some of these same SNPs on mouse G6PC1 protein expression (22) suggests that several of these SNPs differentially affect the stability of mouse and human G6PC1. For example, Ser34Phe, Trp63Arg, Thr111Arg, and Tyr209Ser had limited effects on mouse G6PC1 protein expression (22) but markedly reduced human G6PC1 protein expression (Tables 1 and 2). A direct, simultaneous comparison of the effects of these SNPs on mouse and human G6PC1 protein expression broadly replicated these observations (Fig. 12) ruling out the possibility that these differences arose due to changes in the 832/13 cell line in the time since our previous analyses and instead suggesting that human G6PC1 is more sensitive to mutations than mouse G6PC1.

### Human biobank studies find no evidence for association between nonsynonymous *G6PC2* SNPs and T2D

We previously searched for evidence for association between G6PC2 and T2D risk in humans using Vanderbilt University's Medical Center (VUMC) BioVU biobank, a DNA biobank linked to a deidentified version of the Vanderbilt electronic health records, called the Synthetic Derivative (SD) (31, 32). These studies took advantage of systematic and efficient approaches that have been developed that involve screening the SD with specific SNPs to identify both novel phenotype-variant associations, referred to as PheWAS, and novel associations with clinical lab data, including plasma hormones/metabolites, referred to as LabWAS (33, 34, 35, 36, 37). For these earlier analyses, we used the *G6PC2* rs560887 SNP that has been shown to affect *G6PC2* RNA splicing (16) and has been linked by GWAS to variations in FBG (13, 14). LabWAS and PheWAS analyses showed that the *G6PC2* rs560887 “A” allele was associated with reduced random blood glucose but not altered risk for T2D, respectively (9). As a positive control, BioVU analyses using the established rs13266634 T2D-associated SNP in the *SLC30A8* gene (38) did detect an association with T2D (9). We hypothesized that an association between the *G6PC2* rs560887 SNP and T2D could have been missed due to the modest effect of this SNP on *G6PC2* RNA splicing (16). We therefore repeated the LabWAS and PheWAS analyses using the Ser30Phe, His177Tyr, Tyr207Ser, Ser324Pro, and Arg283STOP SNPs that are present on the array used to genotype BioVU samples and that have a marked deleterious effect on G6PC2 protein expression (Tables 1 and 2). The effects of these SNPs were analyzed as a combined dataset to compensate for their very low MAF. These analyses showed that reduced G6PC2 protein expression was associated with reduced random blood glucose but not altered risk for T2D (Table 3). When analyzed individually, only rs2232323 (Tyr207Ser) was associated with blood glucose, though at lower significance than observed with the combined dataset (*p* < 5.43E-05).

**Table 3:** Analysis of the association between high impact *G6PC2* SNPs with laboratory analytes and disease phenotypes using electronic health record (EHR)-derived analyses

Gene	SNP	Population	Analyte	Effect Estimate	SE	OR	P	N	Bonferroni	FDR
G6PC2	Combined	Total	Glucose Lab	−0.0184	0.0036	0.98	2.24E-07	68,166	TRUE	TRUE


Gene	SNP	Population	Phecode	Description	Beta	SE	OR	P	N_total	N_cases	N_controls	N_no_snp	Bonferroni	FDR
G6PC2	Combined	Total	250.2	Type 2 Diabetes	−0.003	0.01	0.997	0.78	64,618	9760	54,858	949	FALSE	FALSE

Data show an association between high impact *G6PC2* SNPs and blood glucose but not T2D in the total BioVU population. For each laboratory value, we tested for associations between the median of all lab values for an individual against the number of minor alleles for that individual. All associations were adjusted for covariates median age of record, sex, and EHR-reported race using linear regression. The units for all measurements in any given lab were consistent within the lab (e.g., all blood glucose values were reported in mg/dl). “Phecode” refers to the numerical abbreviation for individual phenotype in the Vanderbilt EHR.

FDR, false discovery rate; N_no_SNP, number of records with a missing predictor; OR, odds ratio; SE, standard error.

## Discussion

This study focused on the effects of 22 human *G6PC2* SNPs, 19 of which affect AAs that are conserved between human and mouse G6PC1 and human and mouse G6PC2 (Fig. 2). Of these 22 variants, 16 were found to decrease human G6PC2 protein expression by more than 50% when expressed in the islet-derived 832/13 cell line (Tables 1 and 2) (Fig. S4). Four of the six variants that had little to no impact on G6PC2 protein expression instead reduced G6PC2 activity (Figs. 7 and 8) (Fig. S4).

We previously only studied the effect of 8 of these 22 variants on G6PC2 protein expression in COS cells. Six were found to markedly reduce G6PC2 protein expression (His177Tyr, Tyr207Ser, Arg293Trp, Pro313Leu, Ser324Pro, and Pro340Leu), whereas two (Gly8Glu and Leu219Val) had little effect (22). These results mirror those observed in 832/13 cells and suggest that G6PC2 protein expression and/or stability are mainly determined by ubiquitous rather than cell-line-dependent factors.

PheWAS and LabWAS analyses have the potential to identify previously unknown associations between an SNP linked to a particular disease or clinical parameter and other diseases/clinical parameters. For example, we recently showed that the “C” allele of the nonsynonymous rs13266634 SNP in the *SLC30A8* gene, which confers a gain of function in the ZnT8 zinc transporter, is associated not only with increased T2D risk and blood glucose, but also with increased risk for hemolytic anemia and decreased mean corpuscular hemoglobin (39). Similarly, we showed that the G6PC2 rs560887 “A” allele, which reduces *G6PC2* expression, is associated not only with reduced blood glucose, but also with increased taurine levels and increased risk for acute pancreatitis (9). Our analysis of the combined effect of the deleterious Ser30Phe, His177Tyr, Tyr207Ser, Ser324Pro, and Arg283STOP SNPs only found an association with reduced blood glucose and no other diseases/metabolites (Table 3), though reduced G6PC2 expression was associated with a trend toward reduced hemoglobin A1c (*p* < 0.0036). The lack of replication of our previous observations of associations between G6PC2 and altered taurine levels and increased risk for acute pancreatitis (9) may be explained by a lack of power given the low MAFs of these nonsynonymous SNPs relative to rs560887. Our results on the effect of the His177Tyr and Tyr207Ser SNPs on G6PC2 protein expression are consistent not only with our previous results in COS cells (22) but also with previous reports from other groups (40, 41, 42). However, with the Leu219Val variant, we see little effect on protein expression (Figs. 3 and 5) (Table 2) in contrast to Mahajan *et al.* (40), who observed decreased protein expression of the Leu219 variant relative to Val219. The explanation for this difference is unclear, but we observe the same result using G6PC2 expressed using the pcDNA3.1D vector in COS cells (22) and the pJPA5 vector in 832/13 (Figs. 3 and 5) (Table 2) and βTC-3 cells (Fig. S5).

Because G6PC2 regulates FBG and FBG affects T2D risk, multiple studies have searched for the expected association between G6PC2 and T2D risk. A few studies have reported weak associations (40, 43, 44), but a comprehensive meta-analysis performed using the rs560887 *G6PC2* SNP identified no association (45). Studies on beta cell-specific *G6pc2* KO mice suggest that trace *G6pc2* expression in peripheral tissues is not biologically important with respect to regulation of FBG, consistent with the predominant expression of *G6pc2* in beta cells (9). We hypothesize that this trace *G6pc2* expression in peripheral tissues is not regulating pathways that are masking the expected association between G6PC2 and T2D risk. Instead, we think the explanation for the lack of an association lies in the fact that G6PC2 regulates the glucose sensitivity of GSIS. This creates a situation in which reduced G6PC2 protein expression lowers FBG without affecting fasting plasma insulin (8, 9, 11, 12). Because this reduced FBG would be associated with unchanged glycolytic flux, we previously speculated (9) that it would therefore also be associated with no change in generation of damaging reactive oxygen species (ROS) that appear sufficient to drive beta cell failure (46, 47), explaining the lack of association with T2D risk. One caveat with this concept is that an association with T2D risk could have been missed because rs560887 has a mild effect on *G6PC2* RNA splicing (16). However, our BioVU analyses using nonsynonymous *G6PC2* SNPs that have a major effect on G6PC2 protein expression/activity still found no association with T2D risk (Table 3). Our data suggest that while a therapy directed at G6PC2 may not affect T2D risk, it would likely still be beneficial in preventing other negative consequences of elevated FBG (48, 49, 50, 51, 52, 53, 54, 55).

## Experimental procedures

### G6PC expression vector construction

The construction of plasmids encoding human G6PC2 (accession number NM_021176) and mouse G6PC2 (accession number NM_021331) in the pcDNA3.1D V5 His-TOPO vector has been previously described (3, 4). Both of these plasmids contain the G6PC2 ORF with the native stop codon replaced with an AAG codon encoding lysine followed by sequence encoding a V5 His Tag. In human G6PC2 a common SNP, rs492594, switches a valine to a leucine at AA219 (4). In this study, the human G6PC2 sequence designated as WT contained a leucine at AA219.

A human G6PC1 cDNA (Accession number BC130478; IMAGE clone number 40146509) was purchased from Transomic technologies. This plasmid was used as the template in a PCR reaction with the following primers to generate a human G6PC1 V5 His Tag pcDNA3.1D plasmid:

Sense strand: 5′-GG GGTACC GAGCTC GGATCC AGTACCCTT CACC ATG GAG GAA GGA ATG AAT-3′; Kpn I, Sac I, BamH I restriction enzyme sites, consensus Kozak, and start codon sequences, respectively, are underlined.

Antisense strand: 5′-CCG CTCGAG CG GCC GCC ACT GTG CTG GAT ATC TGC AGA ATT GTC TTG ACC CTT CAA CGA CTT CTT GTG CGG-3′; Xho I restriction enzyme site and a codon encoding a lysine that replaces the native stop codon, respectively, are underlined. The PCR fragment was digested with Kpn I and Xho I and ligated into Kpn I and Xho I digested pcDNA3.1D V5 His-TOPO.

The pJPA5 expression vector was a generous gift from Dr David Jacobson (Vanderbilt University) (24). This vector was modified by insertion of a polylinker containing the following restriction enzyme sites 3′ of the CMV promoter and 5′ untranslated region sequence (Fig. S2): EcoR I, HinD III, Bgl II, BamH I, EcoR V, Xho I, and Pme I creating the plasmid pJPA5 MOD. Human G6PC1 and G6PC2 V5 His pJPA5 MOD plasmids were generated by subcloning the ORFs plus V5 His Tag sequence from the pcDNA3.1D vectors described above into pJPA5 MOD as Hind III–Pme I fragments.

A human G6PC2 no Tag pJPA5 MOD plasmid was generated using PCR in conjunction with the G6PC2 V5 His pJPA5 MOD plasmid described above as the template and the following primers:

Sense strand: 5′-G GAATTC CACC ATG GAT TTC CTT CAC AGG-3′; EcoR I restriction enzyme site, consensus Kozak, and start codon sequence, respectively, are underlined.

Antisense strand: 5′-CCG CTCGAG TGATCA GCTAGC CTT CTG ACT CTT CTT TCC GCT-3′; Xho I, Bcl I, Nhe I restriction enzyme sites and a codon encoding a lysine that replaces the native stop codon, respectively, are underlined. The PCR fragment was digested with EcoR I and Xho I and ligated into EcoR I and Xho I digested pJPA5 MOD.

A human G6PC2 His Tag pJPA5 MOD plasmid was generated by digesting the human G6PC2 no Tag pJPA5 MOD plasmid with Nhe I and Xho I and ligating the following primers that encode a 6X His Tag:

Sense strand: 5′-CTAGC CAT CAT CAC CAT CAC CAT TAA TAG C-3′; Nhe I compatible end and stop codons, respectively, are underlined.

Antisense strand: 5′-TCGAG CTA TTA ATG GTG ATG GTG ATG ATG G-3′; Xho I compatible end is underlined.

A human G6PC2 V5 His Tag pJPA5 MOD plasmid terminating at AA 282 was generated using PCR in conjunction with the G6PC2 V5 His pJPA5 MOD plasmid described above as the template and the following primers:

Sense strand: 5′-CCC AAGCTT GGTACC GAGCTC GGATCC AGT-3′; HinD III, Kpn I, Sac I, and BamH I restriction enzyme sites, respectively, are underlined. This primer recognizes the polylinker sequence 5′ of the G6PC2 sequence.

Antisense strand: 5′-CCC AAGCTT GATATC TGC AGA ATT GTC TTG ACC CTT GCA GCT CAG GAG GAA CAT-3′; HinD III, EcoR V restriction enzyme sites and a codon encoding a lysine that replaces the stop codon in the Arg283STOP variant, respectively, are underlined. The PCR fragment was digested with HinD III and EcoR V and ligated into HinD III and EcoR V digested G6PC2 V5 His pJPA5 MOD.

A mouse G6PC2 no Tag pJPA5 MOD plasmid was generated using PCR in conjunction with the G6PC2 pcDNA3.1D plasmid described above as the template and the following primers:

Sense strand: 5′-G GAATTC CACC ATG GAT TTC CTT CAT AGG-3′; EcoR I restriction enzyme site, consensus Kozak, and start codon sequence, respectively, are underlined.

Antisense strand: 5′-CCG CTCGAG TGATCA GCTAGC CTT TTT AGT CTT CTT GTC ACC-3′; Xho I, Bcl I, Nhe I restriction enzyme sites and a codon encoding a lysine that replaces the native stop codon, respectively, are underlined. The PCR fragment was digested with EcoR I and Xho I and ligated into EcoR I and Xho I digested pJPA5 MOD. A mouse G6PC2 His Tag pJPA5 MOD plasmid was generated using the same strategy as described for the human G6PC2 plasmid. For all the plasmids described above, DNA sequencing was used to verify the absence of secondary mutations in the ORFs and Tag sequences.

Site-directed mutagenesis using the Quikchange II kit (Agilent Technologies) was used to change specific codons in human *G6PC1* and *G6PC2* in the context of the pcDNA3.1D and pJPA5 MOD vectors, respectively. DNA sequencing was used to verify all codon changes and the absence of secondary mutations in the ORF. Two or more independent plasmid preparations were analyzed for each SNP variant described. For those variants that reduced expression, the ORFs plus C terminal V5 His Tag sequence were subcloned into a vector that had not previously been used for site-directed mutagenesis. This controlled for the presence of secondary mutations in the vector backbone that would not have been identified by sequencing. Plasmid purification was achieved by centrifugation through cesium chloride gradients (56).

### SNP databases

Human *G6PC2* SNPs were identified using the dbSNP (http://www.ncbi.nlm.nih.gov/SNP/), UCSC Genome Browser (https://genome.ucsc.edu/) and HumSAVR (http://omictools.com/humsavar-tool) databases.

### Cell culture

Rat islet-derived 832/13 cells (23) were grown in RPMI medium supplemented with 10% (vol/vol) fetal bovine serum, 0.05 mM βmercaptoethanol, 100 U/ml penicillin, and 100 μg/ml streptomycin.

### Protein expression and Western blotting

To determine whether WT and variant human G6PC1 and G6PC2 proteins were expressed at similar levels, plasmids (3 μg) encoding these proteins were transfected into semiconfluent 832/13 cells in 3.5 cm diameter dishes using the lipofectamine reagent (InVitrogen) as previously described (57). Cells were then incubated for 18–20 h in serum-containing medium before harvesting using trypsin, pelleting at 3500*g* for 1 min at room temperature, washing in PBS, and resuspending in 50 mM Tris, pH 8.0, 150 mM NaCl, 0.58 mM PMSF, and 1% NP-40. The Pierce BCA Protein Assay kit (Thermo Fisher Scientific) was used for protein quantitation. Cell extracts (10 μg) were electrophoresed on 10% SDS–polyacrylamide gels and the proteins transferred to a PVDF membrane (PerkinElmer) or nitrocellulose membrane (InVitrogen) in the case of Ponceau S staining. Protein expression was then determined by immunoblotting using the following antibodies: (1) a primary anti-beta actin mouse monoclonal antibody (1:10,000, Sigma) with a secondary anti-mouse horseradish peroxidase (HRP) antibody (1:10,000, Promega); (2) a conjugated mouse monoclonal anti-V5-horseradish peroxidase (HRP) antibody (1:100–1:5000, InVitrogen); or (3) a primary 6x-His tag mouse monoclonal antibody (1:2000, InVitrogen) with a secondary goat anti-mouse IgG2b HRP antibody (1:2000, Invitrogen). HRP activity was assayed using the Pierce ECL reagent (Thermo Fisher Scientific), and Ponceau staining and/or beta actin expression were used as loading controls. Protein expression was quantified by scanning V5 or 6x-His and actin signals on PVDF membranes using a Molecular Imager Gel Doc XR+ System with Image Lab software (BioRad). Images were saved as jpg files and quantified using ImageJ. The ratio of V5 or 6x-His to actin expression obtained with the variants shown was expressed as a percentage relative to the ratio obtained with WT human G6PC1 or G6PC2.

As previously observed, both the human (20) and mouse (21) G6PC1 expression plasmids generate protein doublets, likely indicating the presence of variable glycosylation (58).

### Isolation of microsomal membranes

Cells in six 3.5 cm diameter dishes were transfected and harvested as described above before resuspension in 1 ml cold 50 mM Tris HCl pH 8.0, 50 mM NaCl, 0.5 mM EDTA, 10% glycerol. Cells were then sonicated for 30 s (3 s pulses with 10 s breaks). Cell debris was removed by centrifuging the homogenate at 7800*g* for 6 min at 4 °C. The supernatant was then diluted approximately threefold with the same buffer before being subjected to a high-speed spin (214,000*g*) for 30 min at 4 °C using a Beckman TLA 100.3 rotor so as to isolate a microsomal membrane fraction, which was then resuspended using 300–400 μl of the same resuspension buffer.

### Measurement of glucose-6-phosphatase (G6Pase) activity *in vitro*

For the measurement of G6PC2 activity, microsomal membranes (75 μl) isolated from control cells or cells transiently transfected with a plasmid encoding G6PC2 were mixed with 2X reaction buffer (75 μl; 58 mM MES, 42 mM Tris, 100 mM NaCl pH 6.6) or the same 2X reaction buffer supplemented with 4 mM G6P. Reactions were incubated in a 30 °C water bath for 30–120 min and then placed on ice for 1 min. Reactions were then quenched using a 12% SDS solution (150 μl) and vortexed. A 1:1 mixture of cold Pi chelating solution (300 μl; 2% ammonium molybdate: 12% ascorbic acid) was added to each reaction before incubation at room temperature for 5 min. A developing solution (450 μl; 80 mM sodium citrate, 150 mM sodium meta-arsenite, 2% glacial acetic acid) was then added to the reaction and incubated at room temperature for 20 min. The absorbance of the solution was then measured using a plate reader at 850 nm. The correspondence of absorbance at 850 nm to the total nmol of Pi released was determined from a Pi standard curve. Pi generation by microsomes from control or G6PC2-transfected cells was determined by calculating the difference in Pi detected in the presence or absence of G6P, thus eliminating background Pi introduced by the reaction buffer and microsomes. G6Pase activity (nmol/min/mg) was determined by dividing the Pi generated by the length of the assay and the mg total microsomal protein (as determined by a BioRad Bradford Assay). Control G6Pase activity was subtracted from G6PC2 G6Pase activity as indicated.

For the measurement of G6PC1 activity, microsomal membranes (10 μl) isolated from control cells or cells transiently transfected with a plasmid encoding G6PC1 were diluted with resuspension buffer (65 μl) and then mixed with 2× reaction buffer as described above. Reactions were incubated in a 30 °C water bath for 10 min and then placed on ice for 1 min before continuing the assay as described above for G6PC2.

### Measurement of glucose-6-phosphatase (G6Pase) activity *in situ*

G6Pase activity was measured *in situ* as previously described (22). Briefly, semiconfluent 832/13 cells in 3.5 cm diameter dishes were cotransfected with 2 μg of a *G6pc1*-firefly *luciferase* fusion gene construct, 0.5 μg of SV40-*Renilla luciferase* (Promega) and 1 μg of an expression vector encoding WT or variant G6PC2, using the lipofectamine reagent (InVitrogen). Following transfection, cells were incubated for 18–20 h in serum-free medium supplemented with 2 or 11 mM glucose. Cells were then harvested using passive lysis buffer (Promega) and both firefly and *Renilla* luciferase activity were assayed using the Dual Luciferase Assay kit (Promega). To correct for variations in transfection efficiency, the results were calculated as a ratio of firefly to *Renilla* luciferase activity.

### Electronic health record (EHR)-based analyses of human research subjects

EHR-based analyses were conducted as previously described (9) using data on human subjects in the Vanderbilt University Medical Center (VUMC) BioVU DNA biobank. Genotyping data in BioVU are linked to the Synthetic Derivative (SD), a deidentified version of the VUMC EHR repository. Methods used to perform phenome-wide association studies (PheWAS) and laboratory value-wide association studies (LabWAS) have been previously published (33, 34, 59). PheWAS data were analyzed using an additive model, whereas LabWAS data were analyzed using an additive generalized linear regression model.

### Statistical analysis

Enzyme activity data were analyzed using either the Student's *t* test: two sample, two-sided test assuming equal variance or using an analysis of variants (ANOVA) test with the Tukey's Honest Significant Difference or Dunnett's Multiple Comparison post-hoc test, as stated. *p* values were as indicated. EHR associations were analyzed with logistic and linear regressions using the PheWAS package in R and the LabWAS package in R. Gender, median age of record, and the first ten Principal Component Markers were included as covariates in the PheWAS and LabWAS regressions. PheWAS and LabWAS results were deemed significant if the *p* value of the association passed a Bonferroni multiple testing correction.

## Data availability

All data described are contained within the manuscript.

## Supporting information

This article contains supporting information.

## Conflict of interest

There is no conflict of interest that could be perceived as prejudicing the impartiality of the research reported.
