# Supplementary material for: Nonsynonymous single-nucleotide polymorphisms in the G6PC2 gene affect protein expression, enzyme activity, and fasting blood glucose
Source: J Biol Chem. 2021 Dec 23;298(2):101534. doi: 10.1016/j.jbc.2021.101534 (PMC8800118; doi:10.1016/j.jbc.2021.101534)
Supplement: Fig. S1 [file mmc1.pdf]

Figure S1

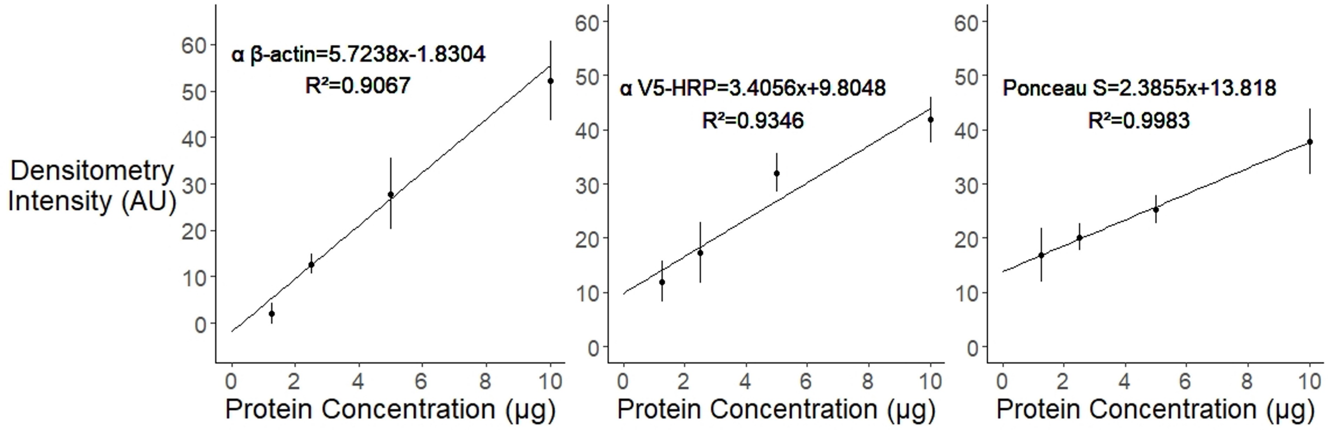

Fig. S1. Quantitation of G6PC2 and Actin Expression and Total Protein Loading.

832/13 cells were transiently transfected with a pJPA5 expression vector encoding human G6PC2 with a C terminal V5 His Tag. Following transfection, cells were incubated for 18-20 hours in serum-containing media. Cells were subsequently harvested and protein expression assayed by Western blotting as described in Experimental Procedures. G6PC expression was assessed using an anti-V5 antibody and equal protein loading was confirmed using both Ponceau staining and measurement of actin expression. The conditions used to assess G6PC2 and actin expression and total protein loading are semi-quantitative. Results show mean data -/+ SD; n=3.
