# Supplementary material for: Nonsynonymous single-nucleotide polymorphisms in the G6PC2 gene affect protein expression, enzyme activity, and fasting blood glucose
Source: J Biol Chem. 2021 Dec 23;298(2):101534. doi: 10.1016/j.jbc.2021.101534 (PMC8800118; doi:10.1016/j.jbc.2021.101534)
Supplement: Fig. S2 [file mmc2.pdf]

Fig. S2

```
pJPA5       1 TAGTAATCAATTACGGGGTCATTAGTTCATAGCCCATATATGGAGTTCCG   50
              ||||||||||||||||||||||||||||||||||||||||||||||||||
pcDNA3      1 TAGTAATCAATTACGGGGTCATTAGTTCATAGCCCATATATGGAGTTCCG   50

pJPA5      51 CGTTACATAACTTACGGTAAATGGCCCGCCTGGCTGACCGCCCAACGACC  100
              ||||||||||||||||||||||||||||||||||||||||||||||||||
pcDNA3     51 CGTTACATAACTTACGGTAAATGGCCCGCCTGGCTGACCGCCCAACGACC  100

pJPA5     101 CCCCGCCCCATTGACGTCAATAATGACGTATGTTCCCATAGTAACGCCAA  150
              |||   ||||||||||||||||||||||||||||||||||||||||||||
pcDNA3    101 CCC--GCCCATTGACGTCAATAATGACGTATGTTCCCATAGTAACGCCAA  148

pJPA5     151 TAGGGACTTTCCATTGACGTCAATGGGTGGAGTATTTACGGTAAACTGCC  200
              ||||||||||||||||||||||||||||||||||||||||||||||||||
pcDNA3    149 TAGGGACTTTCCATTGACGTCAATGGGTGGAGTATTTACGGTAAACTGCC  198

pJPA5     201 CACTTGGCAGTACATCAAGTGTATCATATGCCAAGTACGCCCCCTATTGA  250
              ||||||||||||||||||||||||||||||||||||||||||||||||||
pcDNA3    199 CACTTGGCAGTACATCAAGTGTATCATATGCCAAGTACGCCCCCTATTGA  248

pJPA5     251 CGTCAATGACGGTAAATGGCCCGCCTGGCATTATGCCCAGTACATGACCT  300
              ||||||||||||||||||||||||||||||||||||||||||||||||||
pcDNA3    249 CGTCAATGACGGTAAATGGCCCGCCTGGCATTATGCCCAGTACATGACCT  298

pJPA5     301 TATGGGACTTTCCTACTTGGCAGTACATCTACGTATTAGTCATCGCTATT  350
              ||||||||||||||||||||||||||||||||||||||||||||||||||
pcDNA3    299 TATGGGACTTTCCTACTTGGCAGTACATCTACGTATTAGTCATCGCTATT  348

pJPA5     351 ACCATGGTGATGCGGTTTTGGCAGTACATCAATGGGCGTGGATAGCGGTT  400
              ||||||||||||||||||||||||||||||||||||||||||||||||||
pcDNA3    349 ACCATGGTGATGCGGTTTTGGCAGTACATCAATGGGCGTGGATAGCGGTT  398

pJPA5     401 TGACTCACGGGGATTTCCAAGTCTCCACCCCATTGACGTCAATGGGAGTT  450
              ||||||||||||||||||||||||||||||||||||||||||||||||||
pcDNA3    399 TGACTCACGGGGATTTCCAAGTCTCCACCCCATTGACGTCAATGGGAGTT  448

pJPA5     451 TGTTTTGGCACCAAAATCAACGGGACTTTCCAAAATGTCGTAACAACTCC  500
              ||||||||||||||||||||||||||||||||||||||||||||||||||
pcDNA3    449 TGTTTTGGCACCAAAATCAACGGGACTTTCCAAAATGTCGTAACAACTCC  498

pJPA5     501 GCCCCATTGACGCAAATGGGCGGTAGGCGTGTACGGTGGGAGGTCTATAT  550
              ||||||||||||||||||||||||||||||||||||||||||||||||||
pcDNA3    499 GCCCCATTGACGCAAATGGGCGGTAGGCGTGTACGGTGGGAGGTCTATAT  548

pJPA5     551 AAGCAGAGCTCGTTTAGTGAACCGTCAGATCGCCTGGAGACGCCATCCAC  600
              ||||||||||| | | |  ||            || ||||    | ||||
pcDNA3    549 AAGCAGAGCTC-TCTGGCTAA------------CTAGAGA----ACCCAC  581

pJPA5     601 -GCTGTTTTGACCTCCATAGAAGACACCGGGACCGATCCAGCCTCCGCGG  649
               |||
pcDNA3    582 TGCT----------------------------------------------  585

pJPA5     650 CCGGGAACGGTGCATTGGAACGCGGATTCCCCGTGCCAAGAGTGACGTAA  699

pcDNA3    586 --------------------------------------------------  585
```

```
pJPA5       700 GTACCGCCTATAGAGTCTATAGGCCCACCCCCTTGGCTTCGTTAGAACGC    749
                 |||                             ||||||      | ||
pcDNA3      586 -TAC-----------------------------TGGCTT------ATCG-    598

pJPA5       750 GGCTACAATTAATACATAACCTTATGTATCATACACATACGATTTAGGTG    799
                      |||||                         |||||| |
pcDNA3      599 -----AAATTA-------------------------ATACGACT------    612

pJPA5       800 ACACTATAGAATAACATCCACTTTGCCTTTCTCTCCACAGGTGTCCACTC    849
                 ||||||||                              ||
pcDNA3      613 -CACTATAG------------------------------GG---------    622

pJPA5       850 CCAGGTCCAA-CTGCACCTCGGTTCTATCGATTGAATTCAAGCTTGGTAC    898
                  ||  |||| |||               | |   | | |||||||||||
pcDNA3      623 --AGACCCAAGCTG---------------GCT---AGTTAAGCTTGGTAC    652

pJPA5       899 CGAGCTCGGATCCAGTACCCTTCACCATGGATTTCCTTCACAGGAATGGA    948
                ||||||||||||||||||||||||||||||||||||||||||||||||||
pcDNA3      653 CGAGCTCGGATCCAGTACCCTTCACCATGGATTTCCTTCACAGGAATGGA    702

pJPA5       949 GTGCTCATAATTCAGCA    965
                |||||||||||||||||
pcDNA3      703 GTGCTCATAATTCAGCA    719
```

Fig. S2. Alignment of pcDNA3 and pJPA5 Vector Sequence.

The human G6PC2 open reading frame was sub-cloned as a Hind III – Pme I fragment from the pcDNA3 to the pJPA5 vector. An alignment of the vector sequence 5′ of the Hind III cloning site (red) is shown. The alignment was performed using the EMBOS Water program (https://www.ebi.ac.uk/Tools/psa/). Both vectors share sequences from the cytomegalovirus immediate early enhancer and promoter with the TATA box shown in green. The pJPA5 vector contains additional cytomegalovirus (CMV) 5′ untranslated region sequence shown in blue after the CMV immediate early promoter and a Sp6 promoter shown in orange.
