# Supplementary material for: Nonsynonymous single-nucleotide polymorphisms in the G6PC2 gene affect protein expression, enzyme activity, and fasting blood glucose
Source: J Biol Chem. 2021 Dec 23;298(2):101534. doi: 10.1016/j.jbc.2021.101534 (PMC8800118; doi:10.1016/j.jbc.2021.101534)
Supplement: Fig. S3 [file mmc3.pdf]

Figure S3

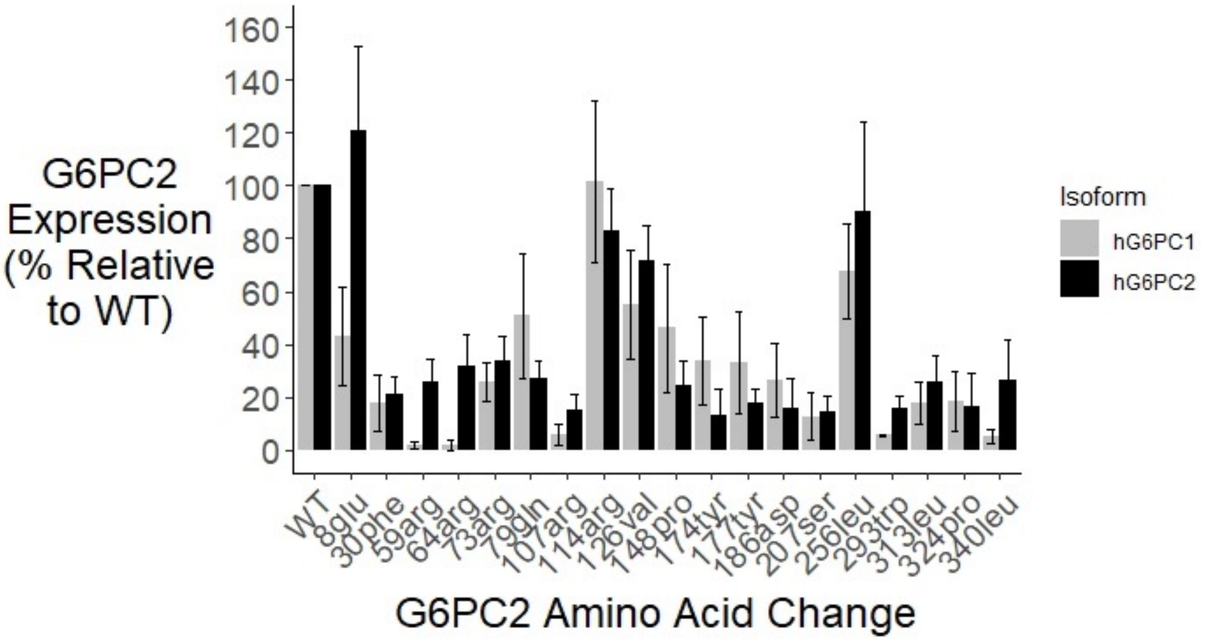

Fig. S3. Analysis of the Effect of Human *G6PC2* SNPs on Human G6PC1 and G6PC2 Protein Expression.

832/13 cells were transiently transfected with pcDNA3 or pJPA5 expression vectors encoding human G6PC1 or G6PC2 with a C terminal V5 His Tag, respectively. Following transfection, cells were incubated for 18-20 hours in serum-containing media. Cells were subsequently harvested and protein expression assayed by Western blotting as described in Experimental Procedures. G6PC2 expression was assessed using an anti-V5 antibody and equal protein loading was confirmed by measurement of actin expression. Results show mean data -/+ SD derived from Figs. 4, 5, 10 & 11.
