# Supplementary material for: Nonsynonymous single-nucleotide polymorphisms in the G6PC2 gene affect protein expression, enzyme activity, and fasting blood glucose
Source: J Biol Chem. 2021 Dec 23;298(2):101534. doi: 10.1016/j.jbc.2021.101534 (PMC8800118; doi:10.1016/j.jbc.2021.101534)
Supplement: Fig. S4 [file mmc4.pdf]

Figure S4

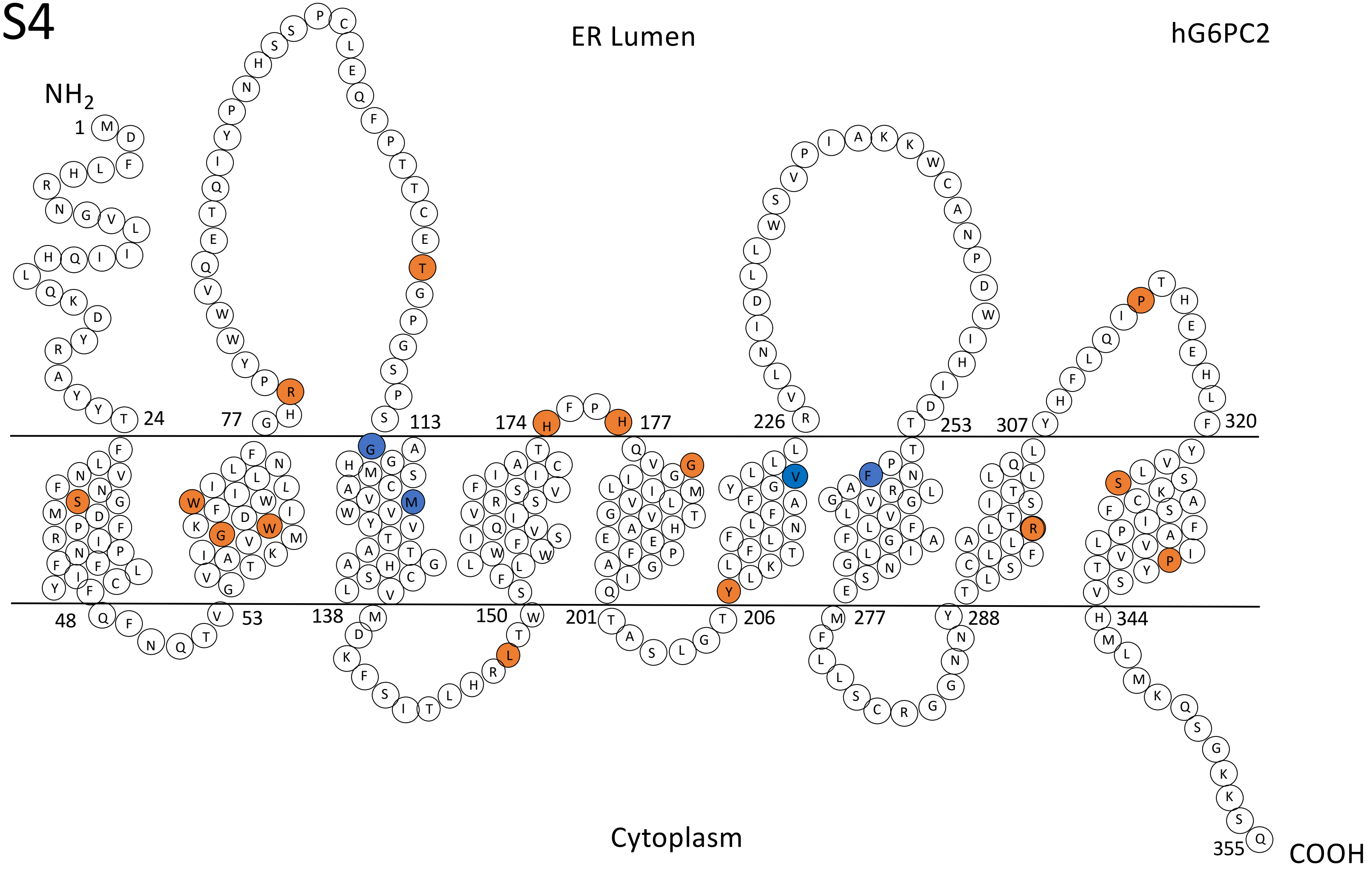

ER Lumen
hG6PC2
NH2
1
24
77
113
174
177
226
253
307
320
48
53
138
150
201
206
277
288
344
355
COOH
Cytoplasm

Figure S4. Location of Amino Acids that Influence G6PC2 Protein Expression or Enzyme Activity.

The TMHMM algorithm (https://services.healthtech.dtu.dk/) (60) was used to predict the location of transmembrane domains in human G6PC1. The predictions made by this program differ from earlier models (4,61). The Figure shows the predicted domains for human G6PC2 based on the location of these domains in human G6PC1, matching the model shown in Figure 2. Residues highlighted in pink represent conserved AAs that are changed by human *G6PC2* SNPs but where mutation in G6PC1 has not been associated with GSD type 1a. Residues highlighted in color represent AAs where the alternate rare allele of specific SNPs result in markedly reduced expression (orange) or activity (blue).
