# Supplementary material for: Nonsynonymous single-nucleotide polymorphisms in the G6PC2 gene affect protein expression, enzyme activity, and fasting blood glucose
Source: J Biol Chem. 2021 Dec 23;298(2):101534. doi: 10.1016/j.jbc.2021.101534 (PMC8800118; doi:10.1016/j.jbc.2021.101534)
Supplement: Fig. S5 [file mmc5.pdf]

Fig. S5

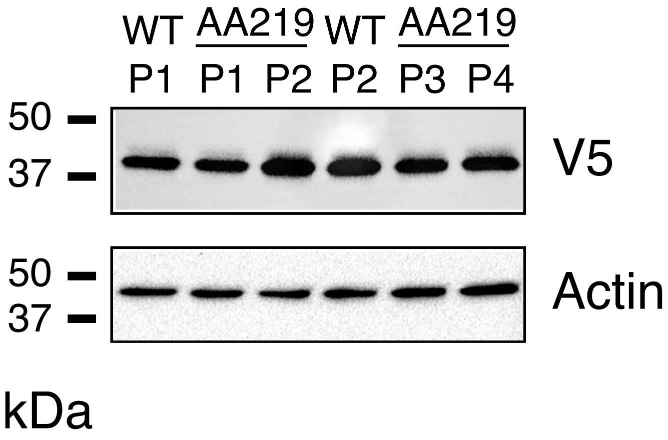

Fig. S5. Analysis of the Effect of the Human G6PC2 Leu219Val SNP on G6PC2 Protein Expression.

βTC-3 cells were transiently transfected with pJPA5 expression vectors encoding human G6PC2 with a C terminal V5 His Tag. Following transfection, cells were incubated for 18-20 hours in serum-containing media. Cells were subsequently harvested and protein expression assayed by Western blotting as described in Experimental Procedures. G6PC2 expression was assessed using an anti-V5 antibody and equal protein loading was confirmed by measurement of actin expression. The blot shows data derived using two independent Leu219 (WT P1, P2) and four independent Val219 plasmids (P1-P4).
